# Supplementary material for: Limiting habenular hyperactivity ameliorates maternal separation-driven depressive-like symptoms
Source: Nat Commun. 2017 Oct 26;8:1135. doi: 10.1038/s41467-017-01192-1 (PMC5658350; doi:10.1038/s41467-017-01192-1)
Supplement: Supplementary file 1 — Supplementary Information [file 41467_2017_1192_MOESM1_ESM.pdf]

Supplementary Information

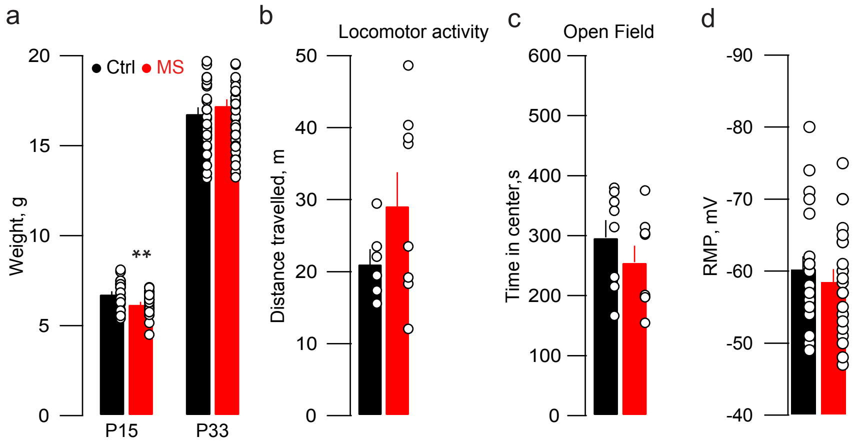

Supplementary Figure 1. Behavioral analysis of MS mice.

(a) Bar graphs and scatter plots depicting weight of mice at P15 and P33 (Ctrl vs MS, n_{mice} = 26 vs 28, P15: unpaired t-test t_{52}=2.98; **p < 0.01, P33; unpaired t-test t_{50}=1.024; p > 0.05). **(b)** Same as **a** but for locomotor activity at P33 (Ctrl vs MS, n_{mice} =6/ 8; distance travelled in 20 min, unpaired t-test t_{12}=1.50; p > 0.05). **(c)** Same as **b** but for activity in the open field (Ctrl vs MS, n_{mice} = 8; time in center, unpaired t-test; t_{14}=1.045 p > 0.05). **(d)** Comparison of resting membrane potential of LHb neurons recorded in Fig. 1e (Ctrl vs MS; aCSF, nmice=6/group; ncells=23/group; unpaired t-test, t_{44}=0.74; p>0.05).

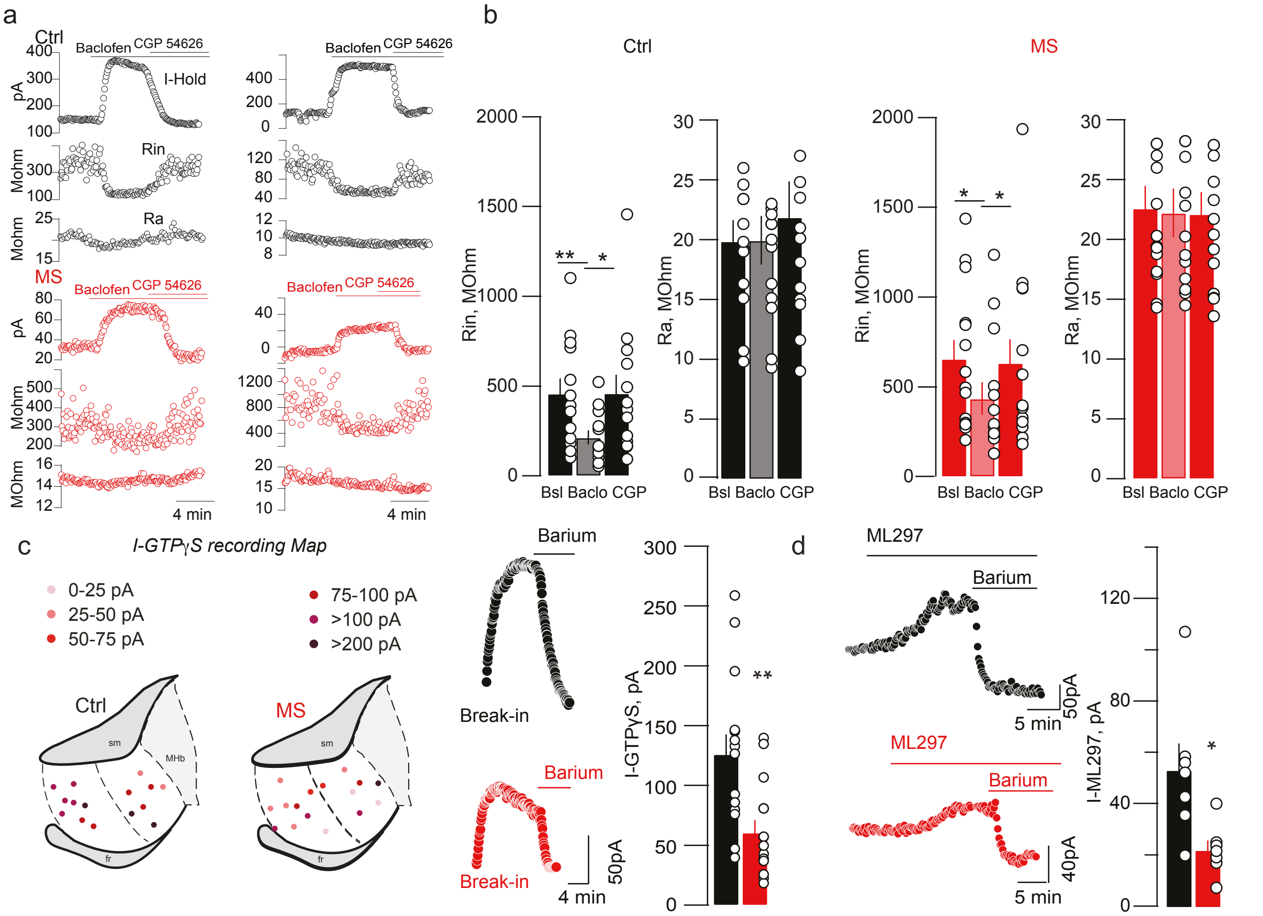

Supplementary Figure 2. MS reduces GABA$_B$-GIRK signaling

(a) Sample traces depicting CGP54626-sensitive I-Baclofen input resistance and access resistance in both experimental groups. **(b)** Bar graph representing Baclofen-mediated changes in input and access resistance in all the experimental groups. (Input resistance: Ctrl mice, baseline vs after baclofen vs CGP54626: n_{mice} = 5; n_{cells}= 13; One-way ANOVA RM; Treatment effect, $F_{(1.532, 18.38)}$ = 11.44, **p<0.01; MS mice: One-way ANOVA RM; Treatment effect, $F_{(1.929, 25.08)}$ = 7.895, **p<0.01) (Access resistance Ctrl mice, baseline vs after baclofen vs CGP54626: n_{mice} = 5; n_{cells}= 13; One-way ANOVA RM; Treatment effect $F_{(2, 36)}$ = 0.239, p>0.05; MS mice: One-way ANOVA RM; Treatment effect, $F_{(2, 39)}$ = 0.017, p>0.05) **(c)** Left, territorial distribution of I-GTPγS (100μM) showing MS-dependent reduction of GIRK signaling throughout the LHb. Right, sample traces, bar graph and scatter plots depicting I-GTPγS in Ctrl and MS mice (I-GTPγS Ctrl vs MS; n_{mice} = 5; n_{cells} = 15; unpaired t-test, t_{28}=3.35; **p < 0.01). **(d)** Sample traces, bar graph

and scatter plots depicting I-ML297 (50μM) in Ctrl and MS mice (I-ML297 Ctrl vs MS; $n_{mice} = 2$; $n_{cells} = 7$; unpaired t-test; $t_{12}=2.84$, *$p < 0.05$).

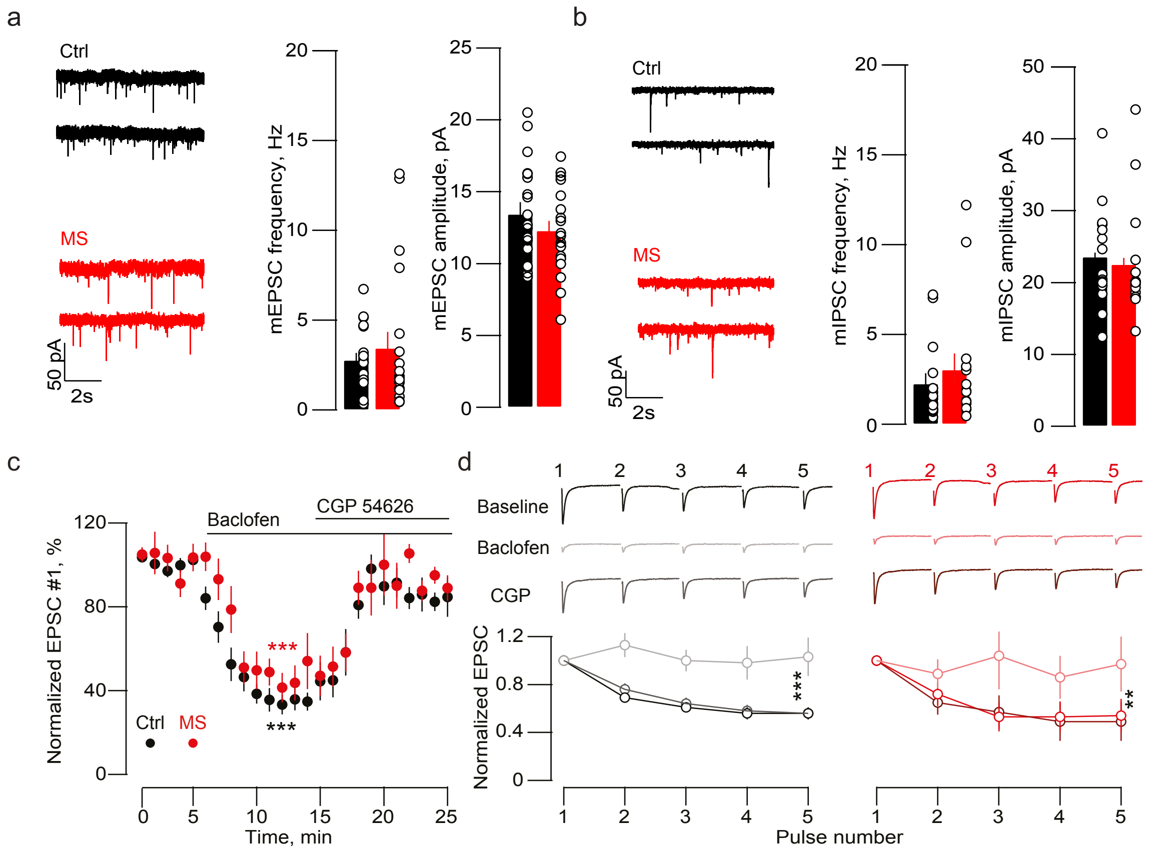

Supplementary Figure 3. MS does not alter synaptic neurotransmission

(a) Sample traces of mEPSCs from LHb neuron of a Ctrl and a MS mice. Bar graphs and scatter plots showing frequency and amplitude of mEPSC for Ctrl and MS mice (Ctrl vs MS, n_{mice} = 8 vs 9, n_{cells} = 20 /group; frequency mEPSC; Kolmogorov-smirnov test, p> 0.05; amplitude mEPSC Ctrl vs MS; unpaired t-test, p > 0.05). **(b)** Same as **a** but for mIPSCs (n_{mice}= 7 /group n_{cells} = 15 /group; frequency mIPSC, Ctrl vs MS; Kolmogorov-smirnov test, p > 0.05; amplitude mIPSC, Ctrl vs MS; unpaired t-test, p > 0.05). **(c)** Timeline showing the baclofen-mediated reduction of evoked EPSC in Ctrl and MS mice (Ctrl vs MS: n_{mice} = 4 and 5; n_{cells} = 7 and 6; two-way ANOVA RM, interaction, $F_{(24,264)}$ = 0.79). **(d)** PPR of EPSCs before, and during baclofen application and subsequent CGP54626 (Normalized EPSC: Ctrl mice, baseline vs after baclofen vs subsequent CGP application for each pulses n_{mice} = 4; n_{cells}= 6; Two-way ANOVA RM; Treatment effect,

$F_{(2,50)}$ = 44.6, ***p<0.001; same for MS mice, n_{mice} = 5; n_{cells}= 6; Two-way ANOVA RM $F_{(2,50)}$ = 15, ***p<0.001).

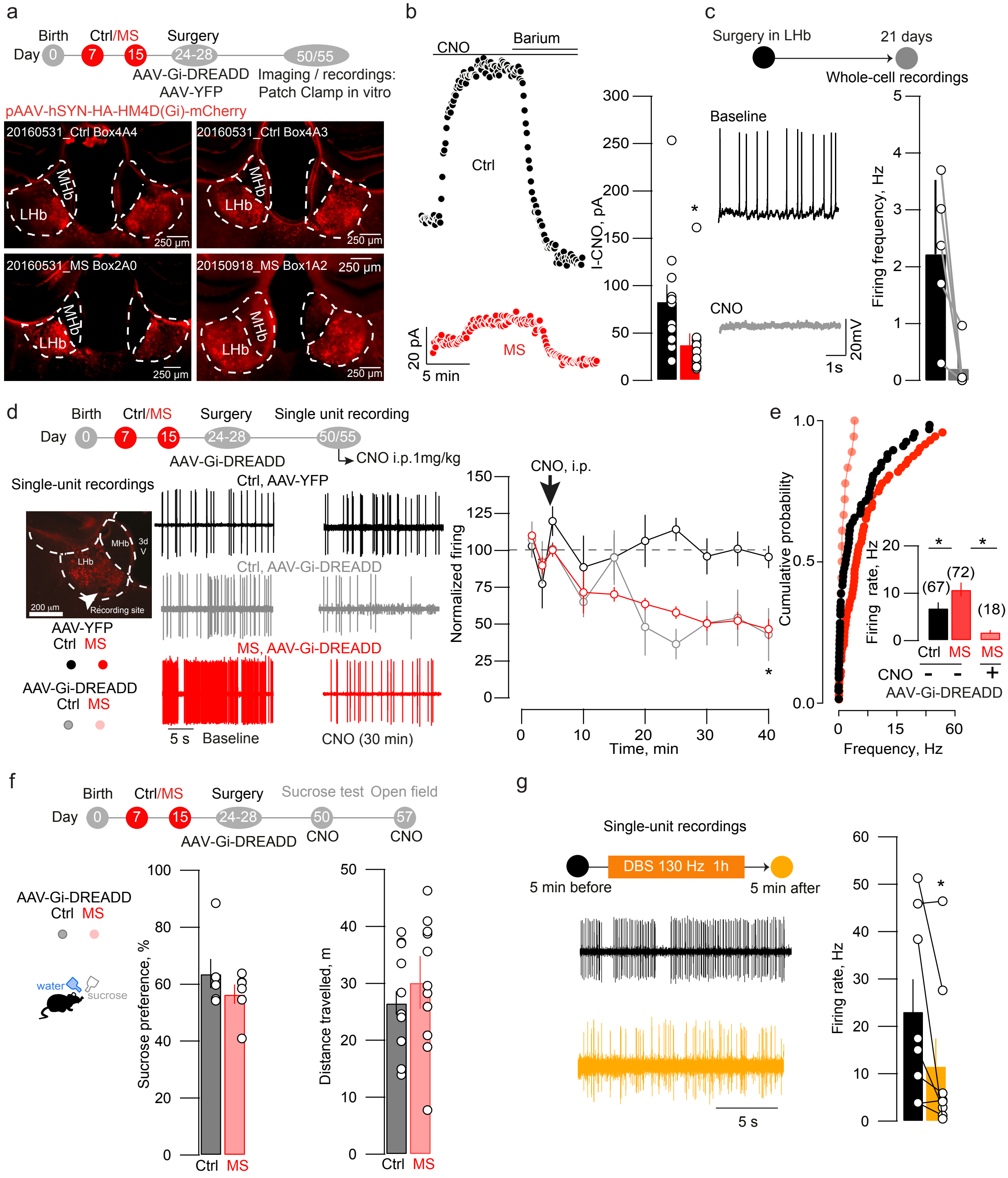

Supplementary Figure 4. Gi-DREADD activation hyperpolarizes neurons and reduces LHb neuronal activity *in vitro* and *in vivo*.

(a) Experimental timeline and representative images (4 different mice) for the site of injection in a coronal slice expressing the Gi-DREADD-mCherry in the LHb. **(b)** Sample traces depicting the CNO-induced current in LHb neurons of Ctrl and MS mice and its reversal by barium (1mM) . Bar graph and scatter plot of the CNO-evoked current amplitude (n_{mice} = 3 /group, n_{cells} = 12 cells /group Ctrl vs MS; unpaired t-test, t_{22}=2.118, *p< 0.05). **(c)** Sample traces depicting the CNO-induced effect on neuronal firing in acute slices in naive mice (Baseline vs post CNO, n_{mice} = 3; n_{cells}= 5; paired t-test, t_4=3.1, *p < 0.05). **(d)** Representative image for Gi-DREADD expression and the site of *in vivo* recording labeled with pontamine sky blue. Sample traces and time course of CNO effect in Ctrl mice expressing AAV-YFP and Ctrl/MS mice expressing AAV-Gi-DREADD. (n_{mice} = 3 vs 4 vs 4, n_{cells} = 3 vs 4 vs 4, Two-way ANOVA, interaction, $F_{(18,72)}$ = 2.15, *p < 0.05). **(e)** Cumlative probability plot depicting the non-normal distribution of baseline firing of LHb neurons recorded in vivo from Gi-DREADD expressing Control (without CNO; $n_{mice/cells}$= 7/67), MS (without CNO; $n_{mice/cells}$= 9/72) and MS with CNO (i.p. 1mg/kg; $n_{mice/cells}$= 4/18) (Kolmogorov-Smirnov test; Control vs MS, D=0.28, **p<0.008; MS vs MS_{CNO} D=0.56 ***p<0.0003; Control vs MS_{CNO}, D=0.35, p>0.05). **(f)** Left**,** effect on sucrose preference in Gi-DREADD Control and MS mice (MS vs Ctrl at baseline n_{mice}= 6 vs 6 , t_{10}=1.17 unpaired t-test, p> 0.05). Right, same for locomotor activity (MS vs Ctrl at baseline n_{mice}= 11 vs 11 , t_{20}=0.82 unpaired t-test, p> 0.05). **(g)** Effect of DBS (1h duration,130Hz, 150μA) on baseline firing recorded in anesthetized mice (n_{mice}=5; n_{cells}=7; paired t-test, t_7=2.412, *p<0.05).
